# Effects of humic substances on Fe(II) sorption onto aluminum oxide and clay

**DOI:** 10.1186/s12932-018-0048-5

**Published:** 2018-01-25

**Authors:** Ying Zhu, Jingjing Liu, Omanjana Goswami, Ashaki A. Rouff, Evert J. Elzinga

**Affiliations:** Department of Earth & Environmental Sciences, Rutgers University, 101 Warren Street, Newark, NJ 07102 USA

**Keywords:** Fe(II), Al, Sorption, Precipitation, Layered double hydroxides, Humic substances, Phyllosilicates, Reducing environments

## Abstract

We studied the effects of humic substances (HS) on the sorption of Fe(II) onto Al-oxide and clay sorbents at pH 7.5 with a combination of batch kinetic experiments and synchrotron Fe *K*-edge EXAFS analyses. Fe(II) sorption was monitored over the course of 4 months in anoxic clay and Al-oxide suspensions amended with variable HS types (humic acid, HA; or fulvic acid, FA) and levels (0, 1, and 4 wt%), and with differing Fe(II) and HS addition sequences (co-sorption and pre-coated experiments, where Fe(II) sorbate was added alongside and after HS addition, respectively). In the Al-oxide suspensions, the presence of HS slowed down the kinetics of Fe(II) sorption, but had limited, if any, effect on the equilibrium aqueous Fe(II) concentrations. EXAFS analyses revealed precipitation of Fe(II)–Al(III)-layered double hydroxide (LDH) phases as the main mode of Fe(II) sorption in both the HA-containing and HA-free systems. These results demonstrate that HS slow down Fe(II) precipitation in the Al-oxide suspensions, but do not affect the composition or stability of the secondary Fe(II)–Al(III)-LDH phases formed. Interference of HS with the precipitation of Fe(II)–Al(III)-LDH was attributed to the formation organo-Al complexes HS limiting the availability of Al for incorporation into secondary layered Fe(II)-hydroxides. In the clay systems, the presence of HA caused a change in the main Fe(II) sorption product from Fe(II)–Al(III)-LDH to a Fe(II)-phyllosilicate containing little structural Al. This was attributed to complexation of Al by HA, in combination with the presence of dissolved Si in the clay suspension enabling phyllosilicate precipitation. The change in Fe(II) precipitation mechanism did not affect the rate of Fe(II) sorption at the lower HA level, suggesting that the inhibition of Fe(II)–Al(III)-LDH formation in this system was countered by enhanced Fe(II)-phyllosilicate precipitation. Reduced rates of Fe(II) sorption at the higher HA level were attributed to surface masking or poisoning by HA of secondary Fe(II) mineral growth at or near the clay surface. Our results suggest that HS play an important role in controlling the kinetics and products of Fe(II) precipitation in reducing soils, with effects modulated by soil mineralogy, HS content, and HS properties. Further work is needed to assess the importance of layered Fe(II) hydroxides in natural reducing environments.

## Introduction

Metal-oxides and clay minerals are important sinks for metal sorbates in soils and sediments, capable of sequestering these elements through various sorption reactions that control their speciation, solubility, and availability for transport and biotic uptake [1, 2]. Metal sorption at mineral–water interfaces may involve various molecular level processes, including adsorption (the formation of mononuclear surface complexes), precipitation (the formation of secondary metal precipitates), and absorption (the incorporation of metal ions into the sorbent lattice) [3, 4]. Operative sorption mechanisms are determined by a variety of factors, including the chemical properties of the metal sorbate and mineral sorbent, the presence of other chemical substances, reaction time, surface loading, pH and ionic strength [1]. Since the mode(s) of metal sorption has a major impact on the kinetics and stability of metal partitioning [1, 3–5], adequate prediction of the behavior and fate of metals in aqueous geochemical systems such as soils requires a thorough mechanistic understanding of prevailing sorption processes.

The precipitation of layered metal hydroxides and phyllosilicates has been identified as a significant sequestration pathway of Zn(II) and Ni(II) in soils with near neutral pH values and higher [6–15]. This is consistent with the results of sorption studies of Co(II), Ni(II), and Zn(II) which have shown that these first-row transition metals readily form layered double hydroxide (LDH) secondary phases during reaction with common soil minerals such as Al-oxides and phyllosilicates at circum neutral pH [16–35]. The metals (Me(II)) precipitate as mixed Me(II)–Al(III)-LDH phases, which consist of brucitic Me(OH)_2_ layers in which up to 1/3 of Me(II) has been substituted with Al(III) derived from weathering of the mineral substrate. The positive layer charge of the substituted sheets is balanced by anions (e.g., nitrate, bicarbonate, sulfate, chloride) coordinated along the basal surfaces [10, 11]. The mechanisms of Me(II)–Al(III)-LDH precipitation during Me(II) sorption are still under debate [36], and may involve various processes, including surface-catalyzed hydrolysis and nucleation of Me(II), Me(II)-promoted dissolution of the mineral sorbent, and heteroepitaxial growth of Me(II)-LDH at or away from the surface of the mineral sorbent. An in-depth discussion is provided in the recent review of Siebecker et al. [36].

Recent studies into the modes of Fe(II) sorption onto Al-oxide and (phyllo)silicate minerals under anoxic conditions have revealed that this first-row transition metal, too, readily forms layered double hydroxides at pH ≥ 7.0 [37–41]. We observed the rapid and extensive precipitation of Fe(II)–Al(III)-LDH during sorption of aqueous Fe(II) (1–3 mM) onto Al-oxide at pH ≥ 7.0 and onto smectite clay at pH 7.0 and 7.5. In addition, we observed the formation of poorly crystalline Fe(II)-phyllosilicates in amorphous SiO_2_ suspensions at pH > 7.5 and in clay suspensions at pH 8.0 [38]. These secondary Fe(II)-phyllosilicates phases are composed of brucitic Fe(OH)_2_ tri-octahedral layers which have fused with (partially) polymerized Si tetrahedral layers [38]. The precipitation of secondary Fe(II)–Al(III)-LDH and Fe(II)-phyllosilicates may play an important role in the sequestration and speciation of Fe(II) in anoxic and suboxic groundwater and soils, where pH values commonly are in the range 6.5–8.0 [42, 43] and reductive dissolution of Fe(III)-oxides may increase dissolved Fe(II) concentrations to mM levels [44–48].

In a recent study, we demonstrated that the formation of Fe(II)–Al(III)-LDH during Fe(II) sorption onto Al-oxide may be significantly impacted by As co-sorbates [39]. The precipitation of Fe(II)–Al(III)-LDH at pH 7.5 was unaffected by As(III), but slowed down at low and intermediate levels of As(V), and shut down in systems with high As(V) concentrations, where Fe(II) surface complexes formed instead. The interference of As(V) with the precipitation of Fe(II)–Al(III)-LDH was attributed to its strong inner-sphere interaction with the Al-oxide surface, blocking the release of Al that is needed for Fe(II)–Al(III)-LDH precipitation [39]. These results imply that the occurrence of layered Fe(II)-hydroxides in soils and sediments will be affected significantly by the heterogeneous composition of these natural systems.

Natural organic matter, which includes the humified materials referred to as humic substances (HS), is a ubiquitous reactive component of terrestrial and aquatic environments [49, 50]. It is well known that organic compounds may affect metal sorption by forming organo-metal complexes in solution, or by associating with metal sorbates at the mineral surface to form ternary complexes [e.g. 51–56]. Previous sorption studies with Zn(II) and Ni(II) have shown that organics may impact the rate of layered Me(II)-hydroxide precipitation and modify the composition of the precipitation products [55, 57–60]. Yamaguchi et al. [57] found that Ni(II) sorption onto gibbsite at pH 7.5 in the presence of citrate and silicate resulted in the formation of α-Ni(OH)_2_ instead of Ni(II)–Al(III)-LDH, while Ni(II)–Al(III)-LDH precipitation during Ni(II) sorption onto pyrophyllite was strongly reduced in the presence of the organic acids. These inhibitive effects were explained by Ni(II) complexation with the organics in solution, and by inner-sphere surface complexation of the organic acids suppressing release of Al^3+^ from the mineral sorbent [57]. Nachtegaal and Sparks [55] similarly demonstrated that natural humic acids reduce the formation of Ni(II)–Al(III)-LDH while promoting precipitation of α-Ni(OH)_2_ during Ni(II) sorption to kaolinite, and additionally observed the formation of HS-Ni(II) adsorption complexes. Li et al. [60] reported that glyphosate suppressed the precipitation of Zn(II)–Al(III)-LDH during Zn(II) sorption on γ-alumina, and attributed this to the formation of ligand-bridged Zn(II) surface complexes competing with Zn(II) precipitation. These earlier studies with Ni(II) and Zn(II) suggest that humic materials may have a considerable impact on the precipitation of layered Fe(II)-hydroxides in soil environments.

The objective of this work was to assess the effects of natural humic compounds on the sorption of Fe(II) in anoxic Al-oxide and clay suspensions under conditions favorable to the precipitation of secondary Fe(II) phases. We employed a combination of batch kinetic studies and synchrotron-based X-ray absorption spectroscopy analyses to investigate how the kinetics and mechanisms of Fe(II) precipitation are impacted by HS content, HS type, and the relative timing of aqueous Fe(II) and HS introduction. The results of this work will enhance our understanding of the role of natural organic matter on the formation of secondary Fe(II) phases in suoxic and anoxic geochemical environments.

## Materials and methods

### Mineral sorbents and organic materials

The mineral sorbents used in this study were the same as in Zhu and Elzinga [35]: γ-Al_2_O_3_ from Alfa Aesar (Cat. No. 39812), and synthetic mica-montmorillonite from the Clay Minerals Repository (Syn-1; Na_0.024_(Al_4.44_Mg_0.04_Fe_tr_)(Si_6.5_Al_1.5_)O_20_(OH)_4_) [61]. These minerals represent the Al-oxide and smectitic clay minerals which are common in soils. The sorbents have negligible Fe contents, enabling characterization of sorbed Fe(II) by X-ray absorption spectroscopy (see below) without interference from structural Fe. The γ-Al_2_O_3_ was used without pretreatment, while the clay was fractionated by sedimentation to isolate the < 1 µm size fraction, which was saturated with Na^+^, and then dialyzed to removes excess salt and freeze-dried. The specific surface areas measured by N_2_-BET were 70.5 m^2^ g^-1^ and 124.6 m^2^ g^-1^ for the γ-Al_2_O_3_ and clay sorbents, respectively. The organic compounds were Suwannee River Fulvic Acid and Suwannee River Humic Acid standard II from the International Humic Substances Society.

### Anoxic conditions

Due to the sensitivity of aqueous Fe(II) to oxidation by O_2(g)_ at the circumneutral pH value of our experiments, the sorption experiments were conducted in an anaerobic glovebox using the same protocols applied in our previous studies [38, 39].

### Coating procedure

The Al-oxide and clay sorbents used for pre-coated sorption experiments were coated with humic acid (HA) at a level of 1- or 4-wt% (with respect to the mass of mineral) following a procedure modified from Nachtegaal and Sparks [55]. The appropriate amount of humic acid was first dissolved into 60 mL of 0.05 M NaOH, followed by adjusting solution pH to 7.50 with 1.0 M HCl. The HA solution was then mixed with 0.625 g mineral sorbent, and the resulting suspension was titrated to pH 3.50. The HA-mineral mixture was placed on a reciprocal shaker for 2 days, after which time the solid was collected by centrifugation for use in the batch kinetic experiments described below. In contrast to the initial HA solutions (i.e., before mixing with the mineral sorbent), the supernatants were transparent without notable coloring, indicating that most HA had been transferred to the solid phase. This was confirmed by solution analyses with UV–Vis [62, 63] which showed that dissolved HA concentrations were below the detection limit of < 2 mg L^−1^, consistent with > 98% removal of dissolved HA during coating.

### Batch kinetic experiments

Two types of batch systems were studied: (i) experiments using pre-coated sorbents, where we monitored Fe(II) sorption onto clay and γ-Al_2_O_3_ sorbents that had been coated with HA using the procedure described in the previous section; and (ii) co-sorption experiments, where we monitored the sorption of Fe(II) that had been introduced simultaneously with HA or FA to suspensions of “clean” (i.e., non-coated) γ-Al_2_O_3_. In both systems, the mineral suspension density was 5 g L^−1^, the background electrolyte consisted of 0.1 M NaCl buffered to pH 7.50 with 50 mM HEPES, and the initial Fe(II)_aq_ concentration was 2.7 mM.

For the pre-coated experiments, the mineral paste recovered from the coating procedure described in the previous section was washed with background electrolyte, and then suspended in a 10 mL volume of background electrolyte. The suspension was transferred into the glovebox and diluted with anoxic background electrolyte to a suspension density of 5 g L^−1^ γ-Al_2_O_3_ or clay. The suspension was hydrated for 2 days in a loosely capped container to allow gas exchange with the glovebox atmosphere for removal of remaining O_2(g)_. Dissolved Fe(II) was then introduced by addition of the appropriate volume of an anoxic acidified 1 M FeCl_2_ stock solution to achieve an initial Fe(II)_aq_ concentration of 2.7 mM. Clay and γ-Al_2_O_3_ control suspensions (containing no HS) were prepared in the same manner.

In the co-sorption experiments, anoxic suspensions of Al-oxide or clay were spiked with 2.7 mM Fe(II)_aq_, with concurrent addition of 0.2 g L^−1^ aqueous HA or FA corresponding to 4-wt% with respect to the sorbent mass. The HA or FA stock suspensions were prepared by dissolving 25 mg HA or FA in 1.5 mL of anoxic 0.05 M NaOH. Control clay and γ-Al_2_O_3_ samples were prepared as well. These suspensions were prepared side by side and in the same manner as the HS containing samples, but without addition of HS.

Following Fe(II) injection, the suspensions were sampled regularly over the course of 120 days. The reaction vessels were wrapped in aluminum foil to prevent light exposure, and sealed in three zip-lock bags inside the glovebox to prevent accidental exposure to O_2_ during equilibration. For the Al-oxide systems, sampling involved filtering 5 mL aliquots through 0.22 µm nitrocellulose membranes, and acidification of the filtrates with 125 µL of anoxic concentrated HCl. Direct filtration was not feasible for the clay samples due to filter clogging. Instead these were centrifuged using airtight centrifuge tubes, and then transferred back into the glovebox where the supernatants were filtered and acidified. The acidified filtrates were analyzed for dissolved Fe(II) using the ferrozine method [64], and Fe(II) sorption was calculated as the difference between the initial and final Fe(II) solution concentrations. The supernatants of the clay sorption samples were additionally analyzed for dissolved Si using inductively coupled plasma optical emission spectroscopy (ICP-OES). The uncertainties of the Fe(II) and Si solution measurements were estimated as ± 5 and ± 15%, respectively, based on quality control samples included in the analyses.

No visible coloring was observed for any of the supernatants, indicating that dissolved HS levels were low. Measurements of dissolved HS in the filtrates using UV–Vis analysis [62, 63] were problematic, because the analyses require alkaline pH values promoting Fe(II) oxidation. Analyses of non-Fe(II) containing control samples yielded no measurable dissolved HS in pre-coated controls, or in HS-γAl_2_O_3_ and HS-clay sorption samples even at short (< 2 h) sorption times. The detection limit of this method was < 2 mg HS L^−1^, which corresponds to < 1% of the maximum HS concentration in the samples. The vast majority of HS in the sorption samples was therefore partitioned to the solid phase. Ritchie and Perdue [65] report a carboxyl content of 6.6 µeg mg^−1^ and a hydroxyl content of 1.4 µeq mg^−1^ for Suwannee Humic Acid, and carboxyl and hydroxyl contents of 4.8 and 1.4 µeg mg^−1^, respectively, for Suwannee River Fulvic Acid. Based on these values, aqueous HA and FA concentrations at the detection limit of 2 mg L^−1^ yield dissolved organic carboxyl and hydroxyl concentrations of 12.4–16.0 µeq L^−1^. These concentrations are > 2 orders of magnitude lower than the Fe(II) concentration of 2.7 mM used in our sorption systems. We therefore conclude that complexation of dissolved HA and FA with Fe(II)_aq_ was not a significant process in our experiments.

### X-ray absorption spectroscopy (XAS) studies

XAS samples were prepared under the same reaction conditions as described above for the kinetic experiments, with reaction times varying from 7 to 158 days. Sample preparation involved terminating the sorption reaction by separating the mineral solid from the solution through centrifugation, and sealing the wet mineral paste into lucite sample holders with Kapton tape inside the glovebox. Reference Fe(II) compounds used for XAS analysis were: [1] aqueous Fe(II); [2] aqueous Fe(II)-HA; [3] aqueous Fe(II)-FA; [4] nikischerite $\left( {{{\text{NaFe}}^{\text{II}}}_{ 6} {\text{Al}}_{ 3} \left( {{\text{SO}}_{ 4} } \right)_{ 2} \left( {\text{OH}} \right)_{ 1 8} \left( {{\text{H}}_{ 2} {\text{O}}} \right)_{ 1 2} } \right)$, which is a natural Fe(II)–Al(III)-LDH mineral; [5] Fe(II)-reacted clay (pH 8.0) and amorphous silica samples (pH 7.5 and 8.0), where Fe(II) is present as poorly crystalline Fe(II)-phyllosilicates. The Fe(II)-clay and Fe(II)-silica samples are from our previous study [38], which also includes a description of the Fe(II)_(aq)_ and nikischerite references. The Fe(II)-HA and Fe(II)-FA solutions were prepared by mixing 10 mM Fe(II) with 1500 mg L^−1^ HA or FA in the same anoxic background electrolyte as used in sorption samples.

Synchrotron Fe *K*-edge (7112 eV) spectra were collected on beamline X-11A of the National Synchrotron Light Source at Brookhaven National Laboratory, and on beamline 12-BM of the Advanced Photon Source at Argonne National Laboratory. Details of the procedures used to ensure that samples remained anoxic during transport and analysis are described in our previous studies [37–39]. The potential of beam damage was checked by visual inspection of each sample immediately following analysis, and by comparing the normalized first and last scans of each sample. Signs of damage were not observed.

EXAFS data analysis and fitting were conducted with WINXAS3.1 [66], combined with Feff 7.0 [67] and ARTEMIS [68]. The fitting procedure was the same as in our previous studies [35, 36]. Briefly, fitting was carried out in R-space using theoretical backscattering paths of Fe–O, Fe–Fe, Fe–Al, and Fe–Si calculated based on the crystal structures of nikischerite; [69], and Fe-substituted lizardite [a 1:1 hydrous Mg silicate; Ref. 70]. The amplitude reduction factor was set at 1, and a single E_0_ shift value was allowed to vary during optimization. For the Fe(II)–γAl_2_O_3_ sorption samples, three single scattering paths were used: first-shell Fe–O, and second-shell Fe–Fe and Fe–Al. The radial distances (R) and Debye–Waller factors (σ^2^) of second-shell Fe–Fe and Fe–Al were constrained to be the same in order to reduce the number of free parameters, while other variables were allowed to vary. For EXAFS data of samples containing secondary Fe(II)-phyllosilicate fitting included first-shell Fe–O and second-shell Fe–Fe and Fe–Si scattering paths. The coordination number of second-shell of Fe atoms was fixed at 6 consistent with tri-octahedral Fe(OH)_2_ sheets, and the Debye–Waller factors (σ^2^) of second-shell Fe–Fe and Fe–Si were constrained to be the same to reduce the number of free parameters. Error estimates of the XAS fitting parameters are ± 0.02 Å for the radial distance (R) of the first coordination shells, and ± 0.04 Å for the radial distances of longer shells. For coordination numbers (CN), which are correlated to the Debye–Waller factor, the estimated error is ± 25% for the first shell and ± 40% for the longer shells [37, 38].

## Results

### Batch kinetic experiments

**Fig. 1:**
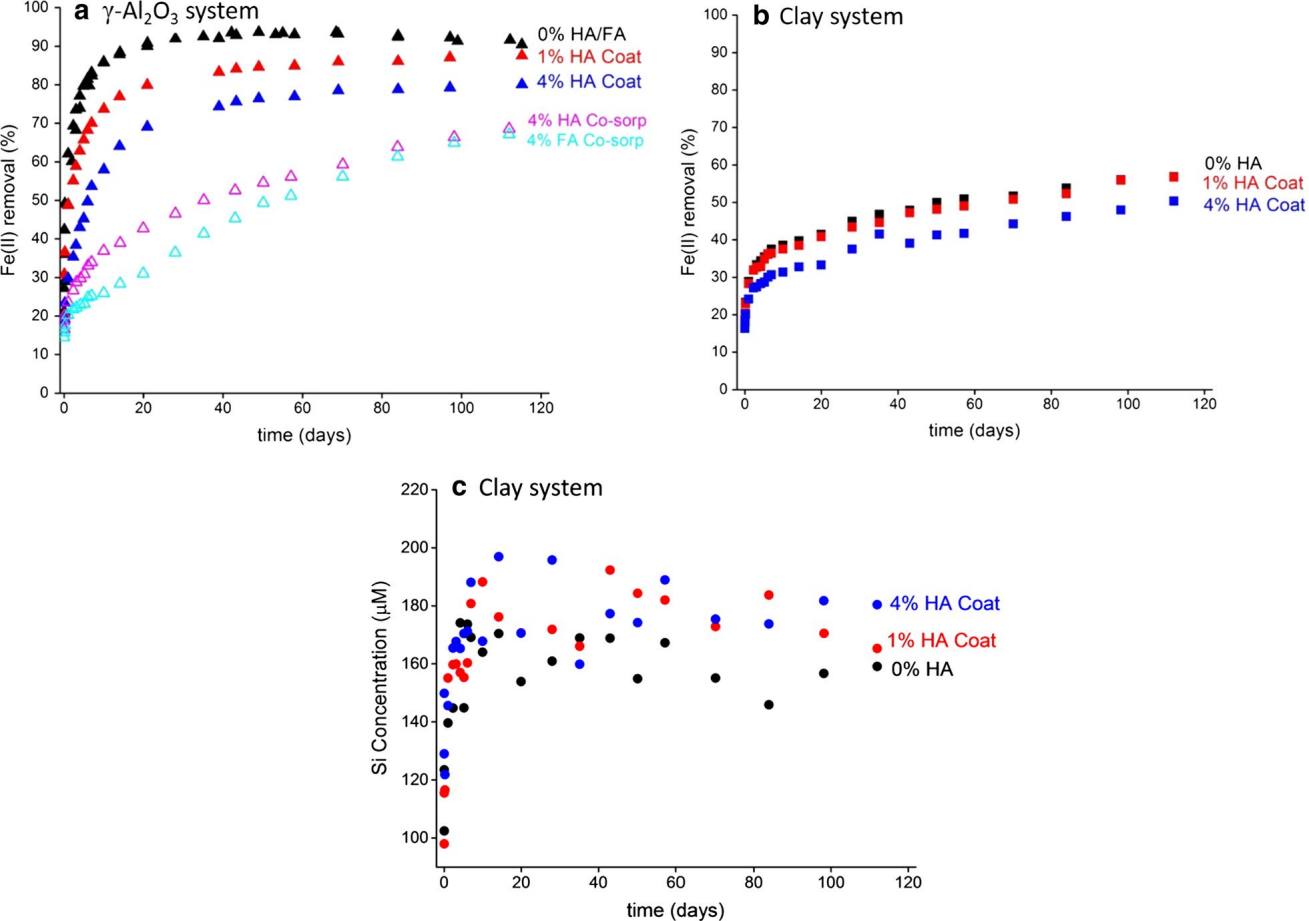
Batch data showing Fe(II) sorption kinetics as affected by HA and FA (1- or 4-wt%) in anoxic γ-Al_2_O_3_ suspensions (**a**) and clay suspension (**b**). **c** The dissolved Si concentration in the clay samples. The mineral suspension density was 5.0 g L^−1^, while the suspension pH was 7.5, and the initial aqueous Fe(II) concentration was 2.7 mM in all systems. The kinetic series labeled 0% HA or FA are control samples of Fe(II) sorption onto γ-Al_2_O_3_ and clay in the absence of HS. The label “coat” refers to experiments using mineral sorbent pre-coated with HA, while “Co-sorp” refers to the co-sorption experiments, where aqueous Fe(II) and HA or FA were introduced simultaneously. The data points cover sorption times ranging from 0.5 h to 118 days

Figure 1 displays the results of the batch experiments, with Fig. 1a showing the kinetics of Fe(II) sorption in the Al-oxide systems and Fig. 1b those in the clay systems; Fig. 1c shows the dissolved Si concentrations in the clay samples. The kinetic Fe(II) sorption patterns vary notably with sorbent type, HS type and concentration, and the timing of Fe(II) addition relative to that of HS (Fig. 1). Differences in reactivity between γ-Al_2_O_3_ and clay towards aqueous Fe(II) are evident from the results of the organic-free control systems. In the γ-Al_2_O_3_ control suspension, Fe(II) sorption proceeds through three different stages: a first stage of rapid Fe(II) uptake during the first hours of reaction, followed by a second stage of slow Fe(II) sorption continuing for ~ 40 days, and finally the equilibrium stage (Fig. 1a). In the organic-free clay sample, the rapid and slow stages of Fe(II) sorption are also observed, but the system does not reach equilibrium in the 3-month time frame of the experiment (Fig. 1b), and the extent of Fe(II) sorption is substantially lower than for γ-Al_2_O_3_ (Fig. 1a). Dissolved Si concentrations in the clay samples increase to values of ~ 160 µM (Fig. 1c). These results are consistent with the findings of our earlier work [38].

The addition of humic substances distinctly modifies the kinetic Fe(II) sorption patterns onto the γ-Al_2_O_3_ and clay sorbents (Fig. 1). For γ-Al_2_O_3_, pre-coating with HA leads to lower Fe(II) sorption relative to the HA-free system across much of the 120 days reaction time frame, and the magnitude of this effect is larger at 4-wt% HA than at 1-wt% HA (Fig. 1a). Of note, however, is that the levels of Fe(II) sorption in the HA-γAl_2_O_3_ systems track to similar end-values as that of the HA-free system, and are within 10% after 120 days (Fig. 1a). It is difficult to ascertain whether equilibrium has been reached in the HA-γAl_2_O_3_ suspensions at this time point, or whether continued slow Fe(II) sorption will further converge Fe(II) sorption in the HA-containing and HA-free systems. The similarity in long-term Fe(II) partitioning in these samples suggests that the effects of HA are mostly kinetic, and that the thermodynamics of Fe(II) sorption are not affected to a major extent by the presence of HA. In the clay samples, pre-coating with 4-wt% HA lowers Fe(II) sorption in the 120-days reaction time frame monitored here, while 1-wt% HA has no discernable impact (Fig. 1b). The kinetics of Fe(II) sorption in the clay samples are notably slower than in the Al-oxide systems, with no evidence of equilibrium attainment after 120 days (Fig. 1b). There are no apparent differences in dissolved Si concentration between the HA-containing and HA-free clay samples (Fig. 1c).

The Fe(II) sorption kinetics in the γ-Al_2_O_3_ systems are not only affected by the amount of HS present (as discussed in the previous paragraph), but also by the timing of Fe(II) addition relative to that of HS, as shown by comparison of the results of the pre-coated versus co-sorption experiments presented in Fig. 1a. In the co-sorption systems, Fe(II) sorption levels are lower than in the pre-coated samples across the 120-days reaction period. In addition, the slow stage of Fe(II) sorption still is clearly ongoing after 120 days in the co-sorption sample, whereas Fe(II) sorption in the pre-coated samples is near or at apparent equilibrium at this time point (Fig. 1a). These observations indicate that co-added HS more strongly interferes with Fe(II) sorption onto γ-Al_2_O_3_ than does pre-added HS. The inhibitive effects of co-sorption are more pronounced for FA than for HA during the first 60 days of reaction, but the difference between FA and HA diminishes at longer sorption times (Fig. 1a). Despite the distinct effects of co-added HS on the Fe(II) sorption kinetics, Fe(II) sorption in the co-sorption systems appears to track towards similar levels as in the pre-coated samples and the HS-free control samples (Fig. 1a), suggesting that the co-addition of HA and FA does not notably change Fe(II) equilibrium partitioning in the γ-Al_2_O_3_ suspensions. Overall, the data presented in Fig. 1 demonstrate that humic substances have a pronounced effect on the kinetics of Fe(II) sorption onto γ-Al_2_O_3_ and clay. The Fe *K*-edge EXAFS data discussed next will be used to assess any changes in the mechanisms of Fe(II) sorption resulting from the presence of HS.

### XAS results

**Fig. 2:**
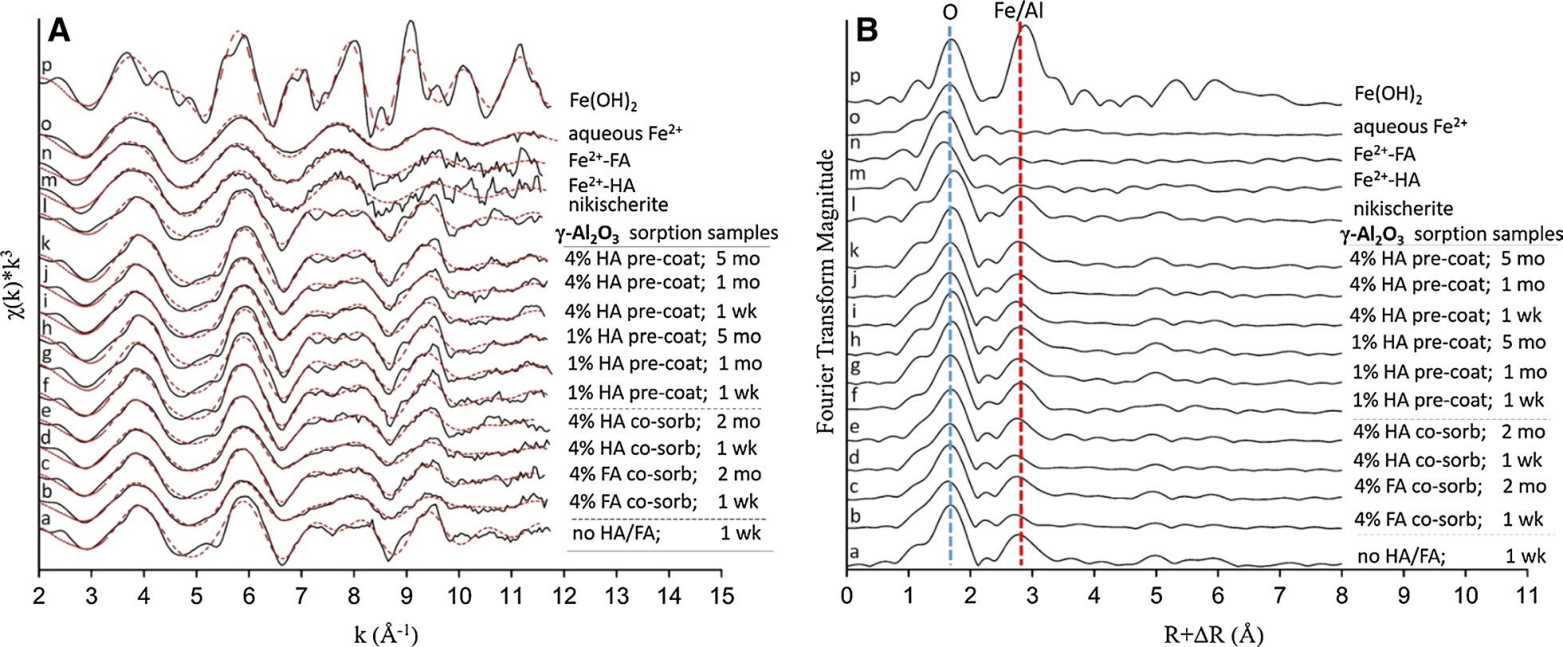
Fe *K*-edge EXAFS results of the Fe references and Fe(II) sorption samples of the γ-Al_2_O_3_ systems where 2.7 mM Fe(II) was reacted at variable HA or FA concentrations for times up to 5 months: **A** *k*^3^-weighted χ spectra; and **B** corresponding radial structure functions (RSFs). Solid and red dotted lines in **A** represent raw and fitted spectra, respectively. The vertical dashed lines in **B** indicate second shell O and metal neighbors. The fit results are summarized in Table 1

**Fig. 3:**
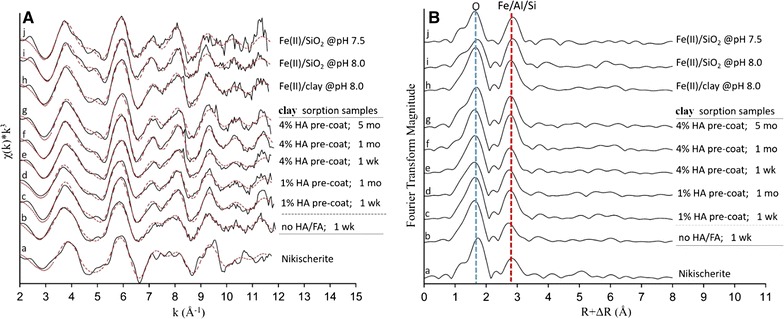
Fe *K*-edge EXAFS results of the Fe references and Fe(II) sorption samples of the clay systems reacted where 2.7 mM Fe(II) was reacted with variable HA concentrations for times up to 5 months: **A** *k*^3^-weighted χ spectra; and **B** corresponding radial structure functions (RSFs). Solid and red dotted lines in **A** represent raw and fitted spectra, respectively. The vertical dashed lines in **B** indicate second shell O and metal neighbors. The fit results are summarized in Table 1

Figures 2 and 3 show the Fe *K*-edge EXAFS data collected for the γ-Al_2_O_3_ (Fig. 2) and clay (Fig. 3) samples where Fe(II) was sorbed at variable HS contents and reaction times. In both figures, panel A shows the raw and fitted *k*^3^-weighted χ spectra, and panel B the corresponding radial structure functions (RSFs) obtained from Fourier transformation of the raw χ data. Also included are the XAS data of the Fe(II) reference compounds. The fit results are summarized in Table 1.

**Table 1:** Fe *K*-edge EXAFS fitting results of Fe(II) reference compounds and sorption samples

Sample			Atomic shell^a^											
			Fe–O			Fe–Fe			Fe–Al			Fe–Si		
			CN	R (Å)	σ^2^ (Å^2^)	CN	R (Å)	σ^2^ (Å^2^)	CN	R (Å)	σ^2^ (Å^2^)	CN	R (Å)	σ^2^ (Å^2^)
*γ-Al*_*2*_ *O*_*3*_ *sorption samples*														
Organic	Type	Time												
–	–	1 week	5.5	2.12	0.008	4.1	3.15	0.010	1.7	3.15	0.010	–	–	–
4% HA	Pre-coat	5 months	5.3	2.12	0.008	3.5	3.15	0.010	1.7	3.15	0.010	–	–	–
4% HA	Pre-coat	1 month	5.3	2.11	0.008	3.2	3.15	0.010	1.9	3.15	0.010	–	–	–
4% HA	Pre-coat	1 week	5.4	2.11	0.009	3.2	3.15	0.010	1.6	3.15	0.010	–	–	–
1% HA	Pre-coat	5 months	5.2	2.13	0.008	3.7	3.15	0.010	2.0	3.15	0.010	–	–	–
1% HA	Pre-coat	1 month	5.7	2.12	0.008	3.6	3.15	0.010	2.0	3.15	0.010	–	–	–
1% HA	Pre-coat	1 week	5.4	2.12	0.008	3.9	3.15	0.010	2.1	3.15	0.010	–	–	–
4% HA	Co-sorb	2 months	5.0	2.12	0.009	3.3	3.15	0.011	1.7	3.15	0.011	–	–	–
4% HA	Co-sorb	1 week	5.9	2.10	0.010	2.0	3.14	0.010	1.0	3.14	0.010	–	–	–
4% FA	Co-sorb	2 months	5.2	2.12	0.009	3.5	3.15	0.011	1.8	3.15	0.011	–	–	–
4% FA	Co-sorb	1 week	5.1	2.10	0.010	2.0	3.14	0.010	1.3	3.14	0.010	–	–	–
*Clay sorption samples*														
Organic	Type	Time												
–	–	1 week	5.9	2.10	0.010	3.9	3.15	0.012	1.8	3.15	0.012	–	–	–
4% HA	Pre-coat	5 months	5.4	2.11	0.010	6.0	3.19	0.012	–	–	–	3.8	3.27	0.012
4% HA	Pre-coat	1 month	5.1	2.10	0.009	6.0	3.20	0.012	–	–	–	4.6	3.30	0.012
4% HA	Pre-coat	1 week	5.4	2.10	0.010	6.0	3.19	0.012	–	–	–	3.8	3.25	0.012
1% HA	Pre-coat	1 month	5.4	2.10	0.011	6.0	3.19	0.012	–	–	–	3.9	3.25	0.012
1% HA	Pre-coat	1 week	5.6	2.10	0.010	6.0	3.19	0.012	–	–	–	4.3	3.26	0.012
*References*														
Nikischerite			5.4	2.14	0.007	3.9	3.15	0.009	2.1	3.15	0.009	–	–	–
Fe(OH)_2_			5.2	2.14	0.005	6.0	3.26	0.006	–	–	–	–	–	–
${{\text{Fe}}^{\text{II}}}_{{\left( {\text{aq}} \right)}}$			5.3	2.12	0.009	–	–	–	–	–	–	–	–	–
Fe^II^-FA_(aq)_			5.5	2.09	0.009	–	–	–	–	–	–	–	–	–
Fe^II^-HA_(aq)_			5.4	2.09	0.009	–	–	–	–	–	–	–	–	–
Fe(II)/clay@pH 8.0			6.1	2.11	0.010	6.0	3.20	0.012	–	–	–	3.4	3.28	0.012
Fe(II)/SiO_2_@pH 8.0			5.3	2.12	0.013	6.0	3.23	0.012	–	–	–	4.0	3.31	0.012
Fe(II)/SiO_2_@pH 7.5			5.2	2.1	0.013	6.0	3.23	0.012	–	–	–	4.0	3.31	0.012

^a^CN is coordination number, R is interatomic radial distance, and σ^2^ is Debye–Waller factor. Error estimates for CN ± 25 and ± 40% for the first and second shells, respectively, and ± 0.02 and ± 0.04 Å for first and second shell R

The RSFs of the Fe(II) sorption samples exhibit a peak at ~ 1.6 Å (uncorrected for phase shift) resulting from backscattering of first-shell O neighbors (Figs. 2B, 3B). Shell fits yield 5.1–6.1 O atoms at a radial distance of 2.09–2.13 Å (Table 1), which are values consistent with an octahedral coordination of Fe(II) by first-shell O ligands, as in the ${{\text{Fe}}^{ 2+ }}_{{({\text{aq}})}}$, β-Fe(OH)_2(s)_ and nikischerite compounds (Table 1) [69, 71–73]. These results confirm the 2+ valence of Fe in our samples, and demonstrate that Fe(II) does not undergo redox changes during sorption or spectroscopic analysis, which is consistent with our earlier work [37–39].

The RSFs of the Fe(II) sorption samples show a second peak at R + ΔR ~ 2.8 Å (Figs. 2B, 3B), which is due to backscattering from Fe atomic neighbors (Table 1) and thus demonstrate the formation of Fe(II) precipitates. The XAS data of the Fe(II)-γAl_2_O_3_ and Fe(II)-clay control samples (containing no HA/FA) are similar to that of nikischerite (Figs. 2, 3; Table 1). Of particular note is the resemblance of the *k*^3^-weighted χ spectra of these control samples to the nikischerite spectrum, including the characteristic “cut-off” beat at 7–8 Å^−1^ that has been identified previously as a signature feature of Fe(II)–Al(III)-LDH [23, 38, 41]. These results therefore demonstrate the precipitation of Fe(II)–Al(III)-LDH phases as the dominant Fe(II) sorption process in both the Fe(II)-γAl_2_O_3_ and Fe(II)-clay control samples, which is consistent with our previous study where we studied Fe(II) interactions with Al-oxide and clay sorbents under similar reaction conditions as applied here [37–39]. These secondary Fe(II) phases consist of positively charged Fe(II)/Al(III) brucite-like sheets with a Fe(II):Al(III) molar ratio of 2:1; the positive layer charge is neutralized by interlayer Cl^−^ anions of the background electrolyte [37].

The EXAFS data and fitting results of the co-sorption and pre-coated γ-Al_2_O_3_ sorption systems demonstrate precipitation of Fe(II)–Al(III)-LDH as the dominant mode of Fe(II) sorption in these systems as well (Fig. 2, spectra b–k; Table 1). This indicates that humic compounds do not change the main mechanism Fe(II) sorption on Al-oxide. This contrasts with the clay systems, where the presence of HA leads to a change in the type of secondary Fe(II) phases formed. In the absence of HA, Fe(II) precipitates as Fe(II)–Al(III)-LDH, as shown by the similarity of the EXAFS data of the Fe(II)-clay control sample (containing no HA) to those of nikischerite (Fig. 3, spectra a, b; Table 1). For the co-sorption samples, however, the Fe XAS spectra resemble Fe(II)-phyllosilicate as represented by the Fe(II)–SiO_2_ and Fe(II)-clay references (Fig. 3, spectra c–j). This is corroborated by the data fits (Table 1) demonstrating slight expansion of the Fe–Fe distance in the HA-containing samples (R = 3.19–3.20 Å) relative to the control clay sample (R = 3.15 Å) and scattering from second-shell Si at 3.25–3.30 Å. These fit results are similar to those of the Fe(II)-phyllosilicate reference samples (Table 1), and agree with the structures of minnesotaite (a 2:1 hydrous iron silicate) and greenalite (1:1) [74–76]. Constructive interference of the signals of second-neighbor Fe and Si enhances overall second-shell backscattering [23, 38], which is observed in the RSFs by an increase in the intensity of the peak at R + ΔR ~ 2.8 Å (Fig. 3B). The XAS results therefore demonstrate that the mechanism of Fe(II) sorption in the clay suspensions changes from Fe(II)–Al(III)-LDH precipitation in the HA-free systems to the formation of Fe(II)-phyllosilicates in the suspensions containing HA-coated clay. Our mechanistic interpretation of these findings is discussed in the next section.

## Discussion

### Impacts of humic substances on Fe(II) sorption onto Al-oxide

Our XAS results demonstrate that Fe(II)–Al(III)-LDHs are the main product of Fe(II) sorption in the Al-oxide suspensions in both the absence and presence of humic substances (Fig. 2; Table 1). Assuming that these phases have the ideal 2:1 molar Fe(II):Al(III) ratio in the octahedral sheets, their aqueous chemical equilibrium can be expressed as [38]:

$$Fe\left( {II} \right)_{{\frac{2}{3}}} Al\left( {III} \right)_{{\frac{1}{3}}} \left( {OH} \right)_{2} Cl_{{\frac{1}{{3_{\left( s \right)} }}}} = \frac{2}{3}Fe^{2 + } + \frac{1}{3}Al^{3 + } + 2OH^{ - } + \frac{1}{3}Cl^{ - } \quad Reaction\; 1$$

Since the experiments were conducted at the same pH (pH 7.5) and in the same electrolyte (0.1 M NaCl), the equilibrium Fe(II) concentrations measured in the batch kinetic studies (Fig. 1a) reflect the thermodynamic stability of the Fe(II)–Al(III)-LDH phases [38]. The Fe(II) solution levels in the HS-free and HS-containing Al-oxide systems presented in Fig. 1a all track towards similar levels (albeit at different rates), suggesting similar stabilities of secondary Fe(II)–Al(III)-LDH in all cases. Unlike the co-sorption experiments, the experiments conducted with HA-precoated Al-oxide appear to be at or near Fe(II) sorption equilibrium after 120 days (Fig. 1a), enabling comparison to the equilibrium [Fe(II)]aq of the HA-free control system to determine whether HA impacts Fe(II)–Al-LDH solubility. The Fe(II) sorption equilibrium in the 1-wt% HA system is very similar to that of the HA-free control system (Fig. 1a), which indicates that HA has essentially no impact on the stability of Fe(II)–Al(III)-LDH in this sample. At 4-wt% HA, Fe(II) sorption after 120 days is lower than in the control (80% vs. 90% of Fe(II) removed; Fig. 1a), which suggests a slightly higher solubility of Fe(II)–Al(III)-LDH relative to the HA free controls. Possible causes may include smaller (nano)particle sizes or lower Al contents of the Fe(II)–Al(III)-LDH phases formed in the HA-containing samples. However, the difference in Fe(II) solubility is small (approximately a factor 2, i.e. well within an order of magnitude; Fig. 1a), which is consistent with the EXAFS data showing no evidence for structural or compositional differences between Fe(II)–Al(III)-LDH formed in the absence and presence of HA (Fig. 2A). We further note that the HA systems may not yet be at Fe(II) sorption equilibrium after 120 days, and that continued slow Fe(II) sorption may lower Fe(II) solubility over longer time scales. Overall, therefore, we conclude that HS have limited, if any, effect on the composition and stability of the Fe(II)–Al(III)-LDH phases formed in the Al-oxide suspensions even at high HS levels.

While HS do not change the end product of Fe(II) precipitation in the Al-oxide suspensions, they do appear to slow down the rate of Fe(II)–Al(III)-LDH formation. Evidence is provided by the XAS results (Fig. 2), from the comparison of the 7-day sorption sample of the HA-free control system (spectrum a, in Fig. 2) versus the 7-day co-sorption samples (spectra b, d in Fig. 2). The intensity of second shell Fe and Al scattering is more pronounced in the HA-free than in the HA-containing sample, indicating more extensive formation of Fe(II)–Al(III)-LDH in the absence of HA at this time point. This correlates with the macroscopic data showing that Fe(II) sorption in the 7 days co-sorption sample is substantially lower than in the 7 days control sample (Fig. 1a). After 2 months, pronounced features of Fe(II)–Al(III)-LDH are seen in the XAS data of the co-sorption samples as well (Fig. 2, spectra c, e) demonstrating ongoing growth of Fe(II)–Al(III)-LDH, which is accompanied by continued removal of Fe(II) from solution (Fig. 1a). From these results, we conclude that the slower Fe(II) sorption rates observed in the batch kinetic studies of the HS-γAl_2_O_3_ systems (Fig. 1a) reflect interference of HS with the growth of secondary Fe(II)–Al(III)-LDH phases, with inhibitive effects modulated by HS content, HS type, and addition sequence.

Several factors may explain the inhibitive effect of HS on Fe(II)–Al(III)-LDH precipitation kinetics. We rule out the formation of aqueous HS-Fe^2+^ complexes as a contributing factor, since dissolved HS concentrations in these systems are negligible relative to the concentrations of Fe(II)_aq_, as noted previously. Instead, the interference of HS with Fe(II)–Al(III)-LDH precipitation in our experiments is due to surface processes involving sorbed HS. Hetero-epitaxial growth of Fe(II)–Al(III)-LDH at the Al-oxide surface may be hindered by HS due to surface masking or due to interactions between HS and Fe(II)–Al(III)-LDH crystallites poisoning their growth. A further possibility is that HS limit the availability of Fe(II) and Al(III) for precipitation as Fe(II)–Al(III)-LDH by forming HS-Fe(II) or HS-Al(III) complexes [77–79]. HS surface complexes may additionally hinder the supply of Al needed for Fe(II)–Al(III)-LDH formation by blocking surface sites of Al dissolution, or by limiting inner-sphere metal-surface interactions which may promote dissolution of the sorbent [80]. In all these scenarios, the reactivity of HS (towards complexation with Fe(II), Al(III), and/or the mineral surface) is the cause of interference with Fe(II) precipitation, which implies that effects will vary with the chemical properties of HS. This is consistent with the difference in impact between FA and HA in the co-sorption experiments with Al-oxide, where FA suppresses Fe(II) sorption more strongly than does HA (Fig. 1a). We attribute this to the higher charge density and the higher content of carboxyl and phenolic functional groups of FA relative to HA [65], enabling more effective interference with precipitation of Fe(II)–Al(III)-LDH.

The mechanism of HS interference with Fe(II)–Al-LDH precipitation in the Al-oxide suspension cannot be conclusively be determined from our data, and it possible that several or all of the mechanisms described above are involved to some extent. However, some constraints can be defined from comparison of the Fe(II) sorption trends in the various experimental systems presented in Fig. 1a. We observe that the suppression of Fe(II)–Al(III)-LDH precipitation by HA is more pronounced in the co-sorption than in the pre-coated experiments (Fig. 1a). This is difficult to explain by a difference in the ability of HA to mask the surface or to block surface sites, because these effects would arguably be more pronounced in the pre-coated systems than in the co-sorption experiments. Competitive complexation of Al^3+^ by HA limiting the availability of Al for incorporation into Fe(II)–Al(III)-LDH is a more plausible explanation. Humic substances have a finite adsorption capacity for Al^3+^, which is determined by the number of organic functional groups available for Al^3+^ complexation [81, 82]. In the pre-coated experiments, HA is pre-equilibrated with the Al-oxide sorbent for 4 days before Fe(II) is introduced (see description of coating procedure in the “Materials and methods” section). As a result, it is likely that the organic molecules are already partially saturated with Al at the onset of Fe(II) sorption in the pre-coated experiments due to partial dissolution of the Al-oxide sorbent. This reduces their competitive impact in these systems relative to the co-sorption experiments where “fresh” (i.e. Al-free) HA is added alongside aqueous Fe(II), explaining why HA interferes less effectively with Fe(II) sorption in the pre-coated samples than in the co-sorption systems (Fig. 1a) . The effect may be compounded by the fact that the functional groups of natural HS exhibit a range of affinities [82], so that the functional moieties most reactive towards Al^3+^ are affected most by pre-equilibration with Al-oxide.

Additional evidence for the importance of Al complexation with HS as an interfering factor slowing Fe(II)–Al(III)-LDH formation in the Al-oxide suspensions is provided by comparison of the results of the HA and FA systems. The Fe(II) sorption kinetic curves of the HA and FA co-sorption experiments start out different, with substantially lower amounts of Fe(II) sorbed in the presence of FA than FA during the first 60 days of sorption (Fig. 1a). We attribute this to the higher density of carboxyl and phenolic groups of FA relative to HA as discussed above. At longer reaction times, however, the effect diminishes and the Fe(II) sorption curves of the HA and FA systems converge (Fig. 1a), suggesting that the difference in reactivity between HA and FA becomes less significant with time. This is inconsistent with surface site blocking or surface masking as the main cause of HS interference with Fe(II) precipitation. A more plausible explanation is that the organic functional groups become saturated with Al^3+^ over time, effectively eliminating the difference in reactivity between HA and FA that dominates the impacts on Fe(II) sorption observed during the earlier sorption stages. Spectroscopic data directly characterizing the speciation of HA and FA in these systems would be required to validate this mechanism and to provide more details of the mechanisms of HS interference with Fe(II)–Al(III)-LDH precipitation.

### Impacts of humic substances on Fe(II) sorption onto clay

In the clay sorption systems, the presence of a HA coating on the clay surface causes a change in the mineralogy of the main Fe(II) sorption product from Fe(II)–Al(III)-LDH to Fe(II)-phyllosilicate. The XAS results show that the Fe(II)-phyllosilicate phase is similar to that formed during Fe(II) sorption onto amorphous silica at pH 7.5 (Fig. 3), which suggests that it contains little or no structural Al. This is consistent with complexation of Al^3+^ by HA limiting its availability for incorporation into secondary Fe(II) precipitates, as suggested for the Al-oxide systems discussed in the previous section. The impact of HA on the composition of the secondary Fe(II) phases formed in the clay systems is a distinct difference with the γ-Al_2_O_3_ sorption systems, where only Fe(II)–Al(III)-LDH forms (Fig. 2). The presence of dissolved Si in the clay suspension (Fig. 1c) clearly is an important factor in influencing the precipitation of Fe(II) in these systems.

Inhibited formation of Fe(II)–Al(III)-LDH in the presence of HA is consistent with the results of Nachtegaal and Sparks [55], who found that the precipitation of Ni(II)–Al(III)-LDH during sorption onto kaolinite clay at pH 7.5 was prevented when the clay was coated with 5-wt% HA. Unlike Fe(II), however, Ni(II) precipitated as a secondary hydroxide (α-Ni(OH)_2_) in the presence of HA, while Fe(II) forms a Fe(II)-phyllosilicate. These results demonstrate differences in the thermodynamic preferences of precipitation between the two metals, and indicate that the impacts of HA on the precipitation of secondary metal phases vary not only with sorbent type but are metal-specific as well.

Inhibited Fe(II) sorption relative to the control is observed in the sample containing 4-wt% HA but not at 1-wt% (Fig. 1b). This contrasts with the XAS data showing that HA changes the composition of the secondary Fe(II) to Fe(II)-phyllosilicate at both 1- and 4-wt% HA coating levels (Fig. 3). Suppressed Fe(II)–Al(III)-LDH precipitation in the HA-clay suspensions therefore does not necessarily translate into slower Fe(II) sorption rates, which is a notable difference with the γ-Al_2_O_3_ systems described above. The mechanisms behind this observation cannot be conclusively deduced from our current data. Complexation of dissolved Fe(II) by surface-bound organic molecules may partially counteract lower Fe(II) sorption rates resulting from inhibited Fe(II)–Al(III)-LDH precipitation, but there is no evidence in the XAS data for this mechanism (Fig. 3). Instead it appears that inhibited Fe(II)–Al(III)-LDH formation in the presence of HA is countered by the formation of Fe(II)-phyllosilicate. This cannot be explained by major differences in dissolved Si levels between HA-free and HA-containing clay systems (Fig. 1c). Instead, structural factors may be involved. Competitive complexation of Al^3+^ by HA in the HA-clay system may induce formation of Fe(II)-hydroxide octahedral sheets with low Al contents, as observed for the Ni(II)-hydroxide phases formed during Ni(II) sorption onto HA-coated kaolinite [55] and during co-sorption of Ni(II) with small organic acids onto gibbsite [57, 58]. These sheets will be structurally less contracted (i.e. have longer Fe–Fe distances) than the mixed Fe(II)–Al(III) sheets of Fe(II)–Al(III)-LDH [35]. This may be favorable to the formation of Fe(II)-phyllosilicates, because their octahedral layer structure is more expanded than that of Fe(II)–Al(III)-LDH [Table 1; 38, 74–76]. As a result, HA may induce precipitation of Fe(II)–phyllosilicate during Fe(II)-clay interaction at a pH value (pH = 7.5) where formation Fe(II)–Al(III)-LDH would otherwise occur (Fig. 2). Validation of this mechanism will require additional work. An implication of the apparent favorability of Fe(II)-phyllosilicate precipitation in these systems is that complexation of Al^3+^ by HA likely is not a major factor in the slow-down in rate of Fe(II) sorption in the 4-wt% HA-clay samples (Fig. 1b). Surface masking or poisoning by HA of secondary Fe(II) mineral growth at or near the clay surface may instead be involved. The mechanisms of HA interference with secondary Fe(II) precipitation may therefore be different in the Al-oxide and clay systems, further highlighting the importance of mineralogy in influencing Fe(II) precipitation behavior.

## Conclusions

The results reported here demonstrate that humic substances may have a substantial influence on the sorption of Fe(II) in reducing soils and sediments at circumneutral pH. We observe that humic acids and fulvic acids slow down the kinetics of Fe(II)–Al(III)-LDH precipitation during Fe(II) sorption onto Al-oxide by reducing the availability of Al^3+^ for incorporation into the secondary Fe(II) minerals. The thermodynamic stability of the Fe(II)–Al(III)-LDH phases is, however, not affected by the humic substances. In the clay suspension, humic acids not only slow down Fe(II) precipitation at high HA levels, but also change the composition of the Fe(II) precipitates by promoting the formation of Fe(II)-phyllosilicates at the expense of Fe(II)–Al(III)-LDH. These results suggest soil mineralogy as an important modulator of the impacts of humic substances on the precipitation of secondary Fe(II) phases under reducing conditions. The rapid and extensive precipitation of layered Fe(II)-hydroxides observed in our experiments even at high levels of HS suggest that these secondary Fe(II) phases may play an important role in controlling the fate of Fe(II) released in riparian natural systems such as river flood plains and wetland soils. However, the mineralogical complexity of soils [3, 4] and the diversity of soil organic matter [83–86] go well below the variability considered in the model systems studied here. Additional work is therefore needed to address the formation kinetics and thermodynamics of layered Fe(II) hydroxide phases in natural environments.
